# The shift towards lower-risk oral food challenges since the implementation of oral immunotherapy: a retrospective cohort study

**DOI:** 10.1186/s13223-026-01028-y

**Published:** 2026-03-29

**Authors:** Adam P. Sage, Min Jung Kim, Tiffany Wong, Raymond Mak, Stephanie C. Erdle, Lianne Soller, Edmond S. Chan

**Affiliations:** 1 Faculty of Medicine, University of British Columbia, Vancouver, BC Canada; 2 Department of Pediatrics, Division of Clinical Immunology and Allergy, BC Children’s Hospital, Room 1C31B, 4480 Oak Street, Vancouver, BC V5Z 4H4 Canada; 3 British Columbia Children’s Hospital, Vancouver, BC Canada

## Abstract

We previously published data on the safety of oral food challenges (OFCs) at BC Children’s Hospital (BCCH), which demonstrated high rates of anaphylaxis requiring epinephrine (15.6%). However, since the 2019 paper, the introduction of oral immunotherapy (OIT) for treatment of food allergies has been widely adopted at our center, resulting in significant practice changes in terms of when and for which patients to offer an OFC. As a result, we sought to compare outcomes of OFCs between the pre-OIT (2014–2017) and post-OIT (2019–2021) eras. We conducted a retrospective chart review of all OFCs completed 2019–2021 at BCCH (post-OIT, n = 590) and compared this to previously published data from OFCs between 2014–2017 (pre-OIT, n = 353). Relative to the pre-OIT cohort, we found patients undergoing OFCs to have significantly lower odds of positive OFCs (26.3% vs 32.6%, OR = 0.738, *p* = 0.0444). Further, OFCs completed after a period of maintenance OIT were found to have reduced odds of requiring epinephrine (8.31% vs 15.0%, OR = 0.542, *p* = 0.0164). We also observed a low severity in those with positive OFCs, with only 3/49 (6.10%) Grade 4 (severe) reactions. While multifactorial in etiology, we found changes in clinical practice with the adoption of OIT to be associated with a significant improvement in safety of OFCs preformed at our institution.

## Background

Oral food challenges (OFCs) are considered the gold-standard of food allergy diagnosis [1]. However, OFCs carry a risk of anaphylaxis, for which currently available data are mixed: Akuete et al. reported 14% of OFCs from 2008–2013 as positive with only 2% having anaphylaxis and 1% requiring epinephrine, compared to Perry et al. who reported 43% positive with 11% requiring epinephrine [2, 3]. As the only tertiary care allergy centre in BC and Yukon, the BC Children’s Hospital (BCCH) Allergy Clinic receives a substantial number of referrals each year, many of which are second-opinion referrals for high-risk OFCs that cannot be done in the community [4]. Considering this, we previously reported an elevated rate of anaphylaxis relative to other Canadian centers, with a 32.6% positivity in OFCs from 2014–2017, 15.6% resulting in anaphylaxis, and 15.0% requiring epinephrine [5].

Since 2019, a major paradigm shift in our clinic has been the implementation of Canadian oral immunotherapy (OIT) guidelines recommending OIT as a treatment option for IgE-mediated food allergy [6]. Data from the Canadian Preschool Peanut Oral Immunotherapy (CPP-OIT) Group suggest that OIT is safe and effective when performed in preschoolers < 6 years of age, with 4.06%, 2.17%, and 3.57% of patients requiring epinephrine for peanut, tree-nut, and sesame OIT respectively, predominantly during the buildup phase (epinephrine was seldomly required during the maintenance phases or exit OFCs) [7–9].

As a result, children are more likely to be initiated immediately on OIT following initial skin prick (SPT) and serum IgE (sIgE) testing, whereas previously, these same children would undergo confirmatory OFCs in order to assess severity and inform recommendations on avoidance. For instance, an older child with a suspected milk allergy and positive SPT as an infant, foregoing a baked milk OFC and instead commencing on oral or sublingual immunotherapy (SLIT). Thus, we hypothesized that the early initiation of formal or modified OIT regimens in favour of high-risk OFCs, has reduced anaphylaxis rates at our institution.

## Methods

We first extracted available data from a previous study (ENHNO) of children who underwent OFCs at BCCH from 2014–2017, prior to the adoption of OIT at our institution [5]. We then performed a retrospective review for a cohort of patients < 18 years old who presented to the BCCH Allergy Clinic for OFCs from January 1, 2019, to December 31, 2021. Cases were identified and screened via review of electronic medical records and detailed clinical and demographic information was extracted. Those undergoing OFCs for FPIES were excluded. We compared outcomes of OFCs in the pre-OIT (2014–2017) and post-OIT (2019–2021) cohorts. Statistical analyses were conducted in RStudio Version 2022.02.1 + 461. As this was a quality improvement project, it qualified for waiver for ethics from University of British Columbia/BC Children’s Hospital Research Ethics Board.

## Results

A total of 353 OFCs were performed between 2014–2017 (pre-OIT) and 590 OFCs were performed between 2019–2021 (post-OIT) (Table 1). The median age was 5 years in both the pre-OIT [IQR 2–10] and post-OIT [IQR 2–9] cohorts with the majority (63.1%) of our cohort being under the age of six. In our patient population, a significant number had comorbidities of allergic rhinitis (n = 371; 62.8%), atopic dermatitis (n = 301; 51.0%), and asthma (n = 153; 25.9%). In our 2019–2021 cohort, 15.6% of challenges performed after a period of maintenance OIT (“post-OIT” challenges), while 62.4% of OFCs conducted were determined to be “Confirmatory OFCs” (for diagnosis), and 22.0% were “Threshold OFCs” (for reassessment of severity of a known allergy) (Table 2).

**Table 1:** Comparison of OFCs at BCCH Allergy Clinic in 2019–2021 vs 2014–2017

Outcome analysis	2019 – 2021 (post-OIT) N = 590	2014 – 2017 (pre-OIT) N = 353	Odds Ratio (post-OIT: pre-OIT), [CI; p-value]
*Epinephrine requirements in all OFCs, n (% total OFCs)*			
Epinephrine	49 (8.31%)	53 (15.0%)	0.513 [0.332–0.792; 0.00160]
No Epinephrine	541 (91.7%)	300 (85.0%)	
*Outcome, n (% total OFCs)*			
Positive OFC	155 (26.3%)	115 (32.6%)	0.738 [0.547–0.995; 0.0444]
Negative OFC	435 (73.7%)	238 (67.4%)	
*Outcome of Positive OFCs, n (% positive OFCs)*			
Epinephrine	49 (31.6%)	53 (46.0%)	0.542 [0.318–0.919; 0.0164]
No Epinephrine	106 (66.4%)	62 (54.0%)	

Types of food challenges	2019 – 2021 (post-OIT) N = 590	2014 – 2017 (pre-OIT) N = 353
*Food Tested, n (% of total OFCs)*		
Peanut	184 (31.2%)	114 (32.3%)
Tree Nuts	189 (32.0%)	69 (19.5%)
Milk	41 (6.9%)	41 (11.6%)
Eggs	79 (13.4%)	91 (25.8%)
Other	97 (16.4)	38 (10.8%)

Descriptive characteristics of OFCs with anaphylaxis	2019 – 2021 (post-OIT) N = 49	2014 – 2017 (pre-OIT) N = 53
Age (Mean)	6.18	7.45
*Anaphylaxis Grading (World Allergy Organization Criteria)*		
Grade 1 (Mild)	0	n/a
Grade 2 (Mild)	31 (63.3%)	n/a
Grade 3 (Moderate)	15 (30.6%)	n/a
Grade 4 (Severe)	3 (6.10%)	n/a
*Systems involved in reaction*		
Skin	47 (95.9%)	48 (90.1%)
GI	30 (61.1%)	40 (75.5%)
Upper respiratory	29 (59.2%)	35 (66.0%)
Lower respiratory	14 (28.6%)	15 (28.3%)
Cardiovascular	4 (8.2%)	4 (7.6%)
*Reaction severity*		
1 dose of epinephrine	32 (65.3%)	44 (83.0%)
2 doses of epinephrine	11 (22.4%)	6 (11.3%)
3 + doses of epinephrine	6 (12.2%)	3 (5.7%)
Code called	8 (1.3%)	n/a
Sent to ER	19 (3.2%)	n/a
Requirement for ICU admission	3 (0.5%)	n/a

**Table 2:** Description of OFCs at BC Children’s Hospital Allergy clinic in 2019–2021

Characteristic	Total (N = 590) n, (% total)	Anaphylaxis Reaction requiring Epi (N = 49) n, (% total epi)
*Age*		
Median (IQR)	5 (2–9)	5 (2–9)
Age ≤ 6, n (%)	372 (63.1%)	34 (69.4%)
Age > 6, n (%)	218 (36.9%)	15 (30.6%)
*Sex: male, n (%)*		
Female	219 (37.2%)	15 (30.6%)
Male	371 (62.8%)	34 (69.4%)
*Medical History, n (%)*		
Asthma	153 (25.9%)	16 (32.7%)
Atopic Dermatitis	301 (51.0%)	31 (63.3%)
Allergic Rhinitis	120 (20.3%)	11 (22.4%)
Multiple Food Allergies	381 (64.6%)	31 (63.3%)
*Prior Anaphylaxis Reaction History, n (%)*		
Total with 1 + prior severe anaphylaxis reaction	278 (47.1%)	24 (49.0%)
Prior ED visit for anaphylaxis	167 (28.3%)	17 (34.7%)
Prior home epinephrine administration for anaphylaxis	80 (13.6%)	7 (14.3%)
Prescribed EpiPen/Allerject/Twinject	560 (94.9%)	49 (100%)
Previously on OIT	92 (15.6%)	2 (4.1%)
*Prior Allergy Testing, n (%)*		
Elevated sIgE (≥ 14)	69 (10.8%)	15 (30.6%)*
sIgE < 14	304 (51.5%)	24 (49.0%)*
sIgE Not Available	217 (36.8%)	10 (20.4%)
Elevated SPT (average ≥ 8 mm)	113 (19.2%)	18 (36.7%)**
SPT < 8 mm	416 (70.5%)	28 (57.1%)**
SPT Not Documented	61 (10.3%)	3 (6.12%)
*Purpose of OFC, n (%)*		
Confirmatory OFC (confirmation of allergy, confirmation of no allergy)	368 (62.4%)	28 (57.1%)
Threshold OFC (max dose testing, reassessment of known allergy, pre-OIT)	130 (22.0%)	19 (38.7%)
Post-OIT Challenge	92 (15.6%)	2 (4.08%)

*Odds ratio = 18.0 [6.91–48.9]; *p* < 0.001

**Odds ratio = 2.62 [1.31–5.14]; *p* = 0.00411

In 2019–2021, 155 (26.3%) OFCs were positive – defined as discontinuation of OFC prior to reaching the target dose secondary to an adverse reaction (Table 1). Forty-nine (8.31%) OFCs in the post-OIT era resulted in an anaphylactic reaction requiring epinephrine, relative to 53 (15.0%) in the pre-OIT era. Notably, patients in our post-OIT (2019–2021) cohort were less likely to have a positive OFC (OR = 0.738, *p* = 0.0444, 95% CI: 0.547–0.995) or have an anaphylactic reaction requiring epinephrine (OR = 0.513, *p* = 0.00160, 95% CI: 0.332–0.792) relative to our pre-OIT (2014–2017) cohort. Table 3 summarizes the characteristics and follow up plan following a positive OFC that did not require epinephrine.

**Table 3:** Description of Reactions in Positive OFCs Not Requiring Epinephrine in the post-OIT Cohort (2019–2021)

Characteristic	Total (n = 106)
*Severity of Reaction, n (%)*	
No Symptoms (challenge stopped due to parental/behavioral concerns)	4 (3.77%)
Grade 1	78 (73.6%)
Grade 2	24 (22.6%)
*Plan After Positive OFC, n (%)*	
Avoid	30 (28.3%)
“May contain” / Limited diet	9 (8.49%)
Continue OIT	11 (10.4%)
Start OIT	50 (47.2%)
Egg/Milk Ladder	4 (3.77%)
Repeat OFC	1 (0.943%)
Full Diet	1 (0.943%)

As a subgroup analysis of the 2019–2021 cohort (Table 4), we looked specifically at the period of time with the highest proportion of post-OIT OFCs (post March 2020). With the increase in post-OIT OFCs from 7.2% to 27.3% post-March 2020, we saw in association significantly decreased rates of anaphylaxis requiring epinephrine from 6.27% to 2.03% (OR = 0.429, *p* = 0.0147, 95% CI 0.199–0.864) (Table 4).

**Table 4:** Stratified Analyses of 2019–2021 Cohort

Pre vs Post March 2020	OFCs Before Mar 2020	OFCs After Mar 2020	Odds Ratio (post-OIT: pre-OIT), [CI; p-value]
*Epinephrine requirements in all OFCs, n (% total OFCs, n* = *590)*			
Epinephrine	37 (6.27%)	12 (2.03%)	0.429 [0.199–0.864; 0.0147]
No Epinephrine	308 (52.2%)	233 (39.5%)	
*Outcome of OFC, n (% total OFCs, n* = *590)*			
Positive OFC	100 (16.9%)	55 (9.32%)	0.866 [0.579–1.29; 0.500]
Negative OFC	266 (45.1%)	169 (28.6%)	
*Outcome of Positive OFCs, n (% positive OFCs, n* = *155, 100 before Mar 2020)*			
Epinephrine	37 (37.0%)	12 (22.0%)	0.477 [0.203–1.07; 0.0705]
No Epinephrine	63 (63.0%)	43 (78.0%)	

## Discussion

The results of this analysis demonstrate a significant reduction in the risk of anaphylaxis in our OFCs following the introduction of OIT.

Presumably, the decreased in OFC-related anaphylaxis following introduction of OIT mirroring that of our 2019 study, is in part due to the shift to higher-risk patients starting immediately on OIT following initial testing, rather than undergoing diagnostic or “confirmatory” OFCs [9]. The relative proportion of high-risk OFCs necessitated prior to the introduction of OIT in our tertiary care allergy clinic include examples such as that of an adolescent patient who has avoided an allergen since infancy with persistently high sIgE and SPT levels, whereby OFC was the only reliable way of confirming individualized reaction severity.

Similarly, we have observed a shift away from baked milk OFCs for preschoolers towards either OIT for more severe reactions or milk ladders for milder reactions. This was reflected in our data with fewer OFCs for cow’s milk (6.9% vs 11.6%) and egg (13.4% vs 25.8%) allergies conducted between 2019–2021 vs 2014–2017 (Table 1), which coincides with the introduction of Canada’s Milk and Egg Ladders in 2019 [10]. Most preschoolers at our institution with egg and milk allergies are now started on food ladders, or alternatively, OIT if considered to be high-risk [6]. In line with this, we observed both an expected increased proportion of OFCs being performed after a period of maintenance OIT, as well as 61.3% of positive OFCs being started on either OIT or egg/milk ladders after their positive challenge (Table 3). We found rates of anaphylaxis to be low (4.1%, N = 2/49) for OFCs completed after a period of maintenance OIT, compared to confirmatory or diagnostic OFCs; therefore, we know that these challenges tend to be quite safe. Therefore, this trend toward less high-risk “confirmatory” OFCs, and more low-risk post-OIT OFCs has likely contributed to an overall decrease in rates of anaphylaxis associated with OFCs.

## Limitations

Our results demonstrate a significant reduction in anaphylaxis/allergic reactions during OFCs at our institution after the introduction of OIT programs. It is a single-centre study, and our results may not be generalizable to all allergy practices in Canada, particularly community practice, although this presents evidence for broader access to OIT programs. Although we note a lower total number of reactions requiring epinephrine, we observed that a greater percentage of total reactions received 2 or more doses of epinephrine, suggesting that these patients who did react were likely a more severe phenotype. However, is important to note that the severity of reaction is variable and contextually dependent, both before and after the introduction of OIT, where in 2014–2017, 18% of reactions had epinephrine given “just-in-case”, guided by a shared decision between patients, families, and providers [9]. Additionally, as patients who were felt to be “high risk” with convincing SPT and/or sIgE were often initiated on OIT without confirmatory OFCs given the high likelihood of reaction. As a result, we are not able to exclude that some patients may not have had a true IgE-mediated food allergy. Furthermore, as a limitation of our retrospective analysis, missing data may skew the interpretation of some results, such as the high proportion of sIgE values being either not performed or not documented (prior to the switch to electronic medical records), as well as the lack of fully comparable demographic information and the specific details of anaphylaxis reactions being unavailable for OFCs conducted pre-OIT (2014–2017).

## Conclusions

In this single-center study, we show a substantial reduction in rates of anaphylaxis requiring epinephrine following the adoption of OIT. Our results highlight the tangible effect OIT has had on our clinical allergy practice and its impact on patient safety with respect to oral food challenges. While examination of individual practices at each institution is required, we are hopeful that with more widespread adoption, OIT can help enable safer OFC practices amongst community and hospital-based allergists alike.

## Data Availability

The datasets generated and analyzed during the current study are not publicly available due to the nature of their summary of patient chart data, but are available from the corresponding author upon reasonable request.

## Abbreviations

BCCH: British Columbia Children’s Hospital
CI: Confidence Interval
CPP-OIT: Canadian Preschool Peanut Oral Immunotherapy Group
FPIES: Food Protein Induced Enterocolitis Syndrome
IQR: Interquartile Range
OFC: Oral Food Challenge
OIT: Oral Immunotherapy
OR: Odds Ratio
sIGE: Serum Immunoglobulin E
SLIT: Sublingual Immunotherapy
SPT: Skin-Prick Test
